# Temperature and photoperiod changes affect cucumber sex expression by different epigenetic regulations

**DOI:** 10.1186/s12870-018-1490-3

**Published:** 2018-11-06

**Authors:** Yun-Song Lai, Di Shen, Wei Zhang, Xiaohui Zhang, Yang Qiu, Haiping Wang, Xinxin Dou, Sigeng Li, Yuanqi Wu, Jiangping Song, Guanyu Ji, Xixiang Li

**Affiliations:** 1 Institute of Vegetables and Flowers, Chinese Academy of Agricultural Sciences, Beijing, 100081 China; 2 Institute of Pomology & Olericulture, Sichuan Agricultural University, Chengdu, 611130 China

**Keywords:** Cucumber germplasm, Sex expression, Temperature, Photoperiod, DNA methylation

## Abstract

**Background:**

Cucumbers (*Cucumis sativus*) are known for their plasticity in sex expression. DNA methylation status determines gene activity but is susceptible to environmental condition changes. Thus, DNA methylation-based epigenetic regulation may at least partially account for the instability of cucumber sex expression. Do temperature and photoperiod that are the two most important environmental factors have equal effect on cucumber sex expression by similar epigenetic regulation mechanism? To answer this question, we did a two-factor experiment of temperature and photoperiod and generated methylome and transcriptome data from cucumber shoot apices.

**Results:**

The seasonal change in the femaleness of a cucumber core germplasm collection was investigated over five consecutive years. As a result, 71.3% of the 359 cucumber accessions significantly decreased their femaleness in early autumn when compared with spring. High temperature and long-day photoperiod treatments, which mimic early autumn conditions, are both unfavorable for female flower formation, and temperature is the predominant factor. High temperatures and long-day treatments both predominantly resulted in hypermethylation compared to demethylation, and temperature effect was decisive. The targeted cytosines shared in high-temperature and long-day photoperiod treatment showed the same change in DNA methylation level. Moreover, differentially expressed TEs (DETs) and the predicted epiregulation sites were clustered across chromosomes, and importantly, these sites were reproducible among different treatments. Essentially, the photoperiod treatment preferentially and significantly influenced flower development processes, while temperature treatment produced stronger responses from phytohormone-pathway-related genes. Cucumber *AGAMOUS* was likely epicontrolled exclusively by photoperiod while *CAULIFLOWER A* and *CsACO*_*3*_ were likely epicontrolled by both photoperiod and temperature.

**Conclusions:**

Seasonal change of sex expression is a germplasm-wide phenomenon in cucumbers. High temperature and long-day photoperiod might have the same effect on the methylome via the same mechanism of gene-TE interaction but resulted in different epicontrol sites that account for different mechanisms between temperature- and photoperiod-dependent sex expression changes.

**Electronic supplementary material:**

The online version of this article (10.1186/s12870-018-1490-3) contains supplementary material, which is available to authorized users.

## Background

Like many other *Cucurbitaceae* plants, cucumber plants have unisexual flowers and are usually monoecious [1]. According to the ratio of female to male flowers and their distribution, monoecious plants are mainly grouped into normal monoecy, subgynoecy and subandroecy. Cucumbers also have the sexual morphs of gynoecy, gynomonoecy, androecy, hermaphrodity, andromonoecy and trimonoecy, but these morphs are rare.

The female/male flower ratio in cucumbers is largely unstable, which is affected by environment condition change. The effects of light intensity [2], light quality [3], nitrogen and watering stress [4], and mechanical stress [5] have been reported. However, these environmental factors are relatively less decisive than temperature and photoperiod of which effects on cucumber sex expression have been characterized many years ago [2, 4, 6–9]. Essentially, conditions of high temperature and long-day conditions suppress female flower formation depending on the cucumber genotype in the above studies. The phenomenon of environment-dependent sex expression is not only restricted to cucumbers but seem a common feature in higher plants [10].

Despite the same suppressive effect, high temperature and long photoperiod don’t have equal influence on cucumber sex expression. High temperature treatment significantly decreased the female flower node ratio of Chinese Long cucumber (or ‘9930’) as much as 85.6% [11]. This value is much higher than what we observed in long-day treatment (52.9%). Cucumbers are known for their seasonal change in female flower rate. Integration of the conditions of higher temperatures and long days in early autumn might play a role, and the former environment factor seems more decisive. It remains unknown about the interaction between temperature and photoperiod effect.

Ethylene regulation is anticipated to be involved in environment-dependent sex expression of cucumbers because ethylene is the “sex hormone” of cucumbers [12–14]. The genetically identified “sex genes” *female* (*F*) [15–18], *monoecious* (*M*) [19–21], and *androecious* (*A*) [22] encode 1-aminocyclopropane-1-carboxylate synthase (ACS), which mediates the first committed step for ethylene biosynthesis and controls metabolic flux from methionine salvage. Given the critical role of ethylene, the “one-hormone model” law of cucumber sex determination was proposed, and it appears indisputable after many years [23]. As anticipated, there is a diurnal rhythm of ethylene accumulation, and CsACS2 expression in shoot apices of monoecious plants, along with ethylene biosynthesis, is more active under short-day conditions than long-day conditions [24]. Ethylene signaling has been suggested to participate in high-temperature-induced femaleness suppression [9]. Most recently, we proposed that the epiregulation of ethylene-related genes and MADS-box genes might potentially account for temperature-dependent sex expression [11].

Here, we observed the germplasm-wide seasonal change of female flower node rate in consecutive years. Chinese long cucumber ‘9930’ is a typical monoecious line whose sex expression is sensitive to seasonal change. We applied a two-factor treatment of temperature and photoperiod on ‘9930’ and thoroughly compared the effect of photoperiod and temperature on transcriptome and methylome in shoot apices. This study interprets the interaction of high temperature and long-day condition in the suppression of female flower formation in cucumbers.

## Results

### The seasonal plasticity of female/male flower ratios in the cucumber germplasm is related to temperature and photoperiod

The seasonal sex stability of 359 accessions from a cucumber core germplasm collection was assessed for 5 consecutive years (2010–2014) (Additional file 1: Table S1). The core germplasm collection was established previously [16, 25–29]. Because Xishuangbanna cucumbers (*Cucumis sativus* L. var. *xishuangbannanesis*) grow abnormally under regular cultivation condition in Beijing, they were excluded in the field survey. All cucumber materials can be classified into three groups according to the proportion of nodes with pistillate flowers (PNPF) in spring: 158 subandroecy (PNPF< 25%), 191 normal monoecy (25% ≤ PNPF< 75%), and 10 gynoecy/subgynoecy (PNPF≥75%).

**Fig. 1:**
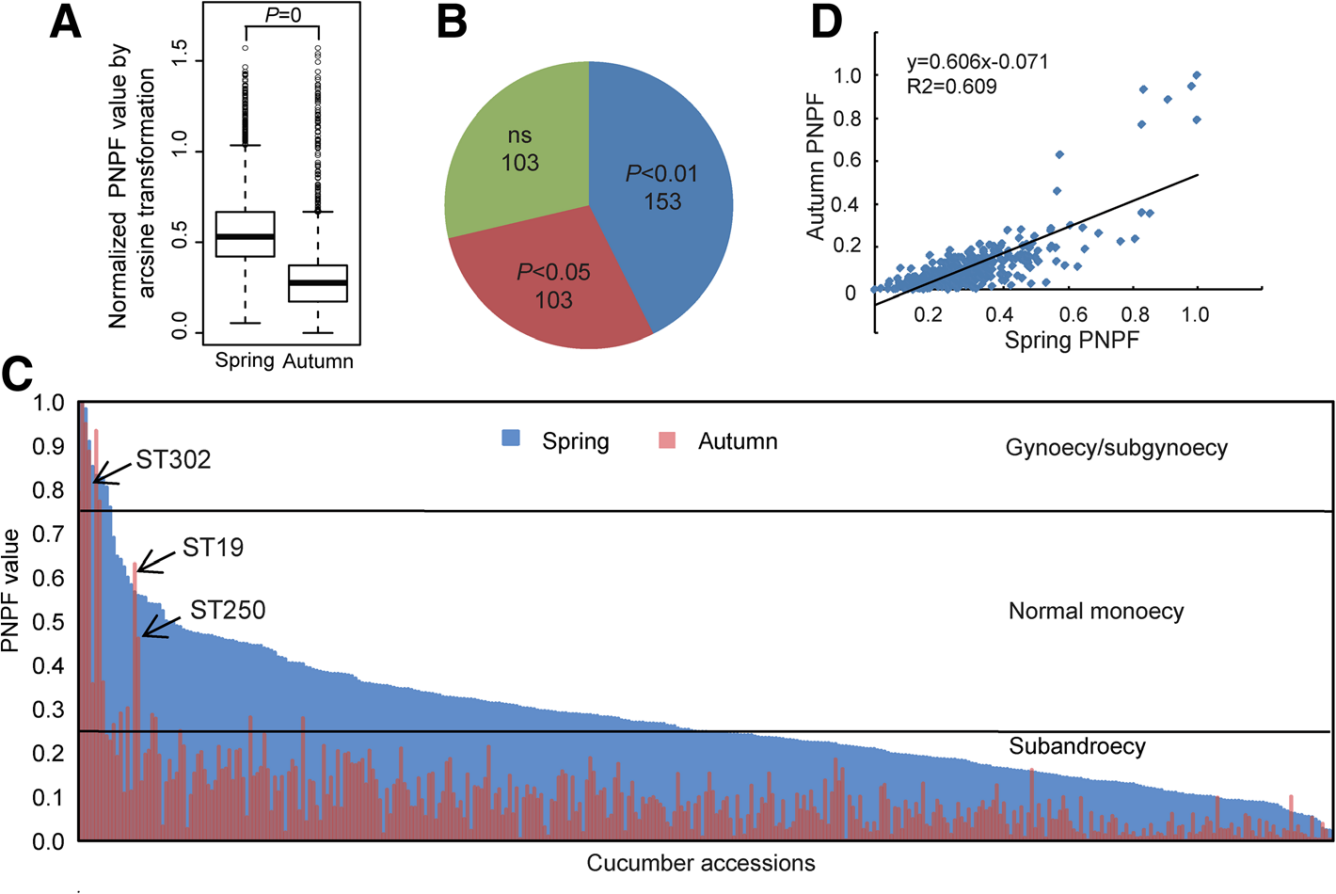
Seasonal change in cucumber femaleness indicated by the proportion of nodes with pistillate flowers (PNPF) value was observed during consecutive years using core germplasm collections. **a** The box plot shows the overall range and distribution of the normalized PNPF value. The bar in the box indicates median value. There is significant difference in the PNPF mean value of the entire germplasm between spring and early autumn in a *t*-test. **b** Number of cucumber accessions showing a significant seasonal change in the PNPF according to a *t*-test. ns, not significant (*P* > 0.05). **c** The distribution of 359 cucumber accessions with various PNPF value in spring and autumn. Instable gynoecious/subgynoecious lines as well as stable normal monoecious lines are exceptions in cucumber germplasm and therefore are highlighted by arrows. **d** The linear relationship between the PNPF in spring and early autumn

Taking all the accessions as a whole, PNPF value of cucumbers grown in early autumn was significantly decreased when compared with that in spring (Fig. 1a). Over 93.3% of the total accessions had PNPF values that decreased by more than 40% in early autumn, among which 71.3% showed a statistical significance (*p* < 0.05) (Fig. 1b). These results indicated that the response of femaleness to seasonal change is a germplasm-wide manner. Nevertheless, there were still 103 accessions showing no significant decrease in the PNPF, among which gynoecious/subgynoecious plants were notably stable except for accession ST302 (Fig. 1c). Notably, two normal monoecious lines numbered ST19 and ST250, which are from Israel and China respectively, show very stable sex expression. Moreover, the PNPF values in the spring and early autumn showed positive linear relationships (Fig. 1d). This finding indicated that a seasonal change did not result in a PNPF change in each cucumber accession to an equal extent. Analysis of variance (ANOVA) for cucumber PNPF value was performed to determine the effect of genotype, season, year and the interaction effects among these factors. As a result, significant differences were observed among genotypes, among years and between seasons (Table 1). There were also significant differences between the interactions among the three factors. As anticipated, genotype and season were the two major factors that contributed to the variance. In nature, temperature and photoperiod are the most important factors in a seasonal shift (Additional file 1: Table S2). Cucumber ‘9930’, ST359, ST360, and ST361 are representative of normal monoecy, subandroecy, Xishuangbanna, and gynoecy group, respectively, and were subjected to treatments of temperature and photoperiod under controlled conditions in incubators. A two-factor ANOVA was performed to assess the major sources of variability in the PNPF value: variety, temperature, photoperiod, and the interaction between them. As a result, significant differences were observed between genotype, between temperature and between photoperiod as well as their interaction (Table 1). Genotype is the biggest source of variance and we further did ANOVA analysis within cucumber accessions. As a result, temperature (ANOVA, fluence = 151.0), photoperiod (fluence = 39.4), and their interaction (fluence = 28.5) were found to have significant effects (*p* < 0.01) on femaleness in ‘9930’. For ST360, only the photoperiod (fluence = 318.7) had a significant effect (*p* < 0.01). There were no significant effects (*p* < 0.01) in ST359 or ST361. These results indicated that the effect of temperature and photoperiod is dependent on genotypes. Temperature effect appears predominant over photoperiod effect for ‘9930’, a typical normal monoecious line.

**Table 1:** ANOVA of PNPF value in cucumbers

Source of variation	df	SS	MS	TSS (%)
Field investigation				
Year	4	3.20	0.800**	0.51
Variety	358	295.00	0.824**	47.15
Season (Year)	5	202.00	40.390**	32.29
Year ×Variety	1432	55.66	0.038**	8.90
Season (Year) × Variety	1790	68.74	0.038**	10.99
Residual	7180	1.02	0.000	0.16
Total	10,769	625.60		
Two-factor treatment of temperature and photoperiod				
Variety	3	7.274	2.425**	28.665
Temperature	1	0.239	0.239**	0.943
Photoperiod	1	0.340	0.340**	1.338
Variety × Temp	3	0.344	0.115**	1.354
Variety × Photo	3	0.215	0.072**	0.848
Temp × Photo	1	0.046	0.046**	0.182
Variety × Temp × Photo	3	0.031	0.010	0.121
Residual	32	0.113	0.004	0.443
Total	48	25.377		

** indicate significance at *P* < 0.01

### Comparison of transcriptome changes in response to temperature and photoperiod treatment

**Fig. 2:**
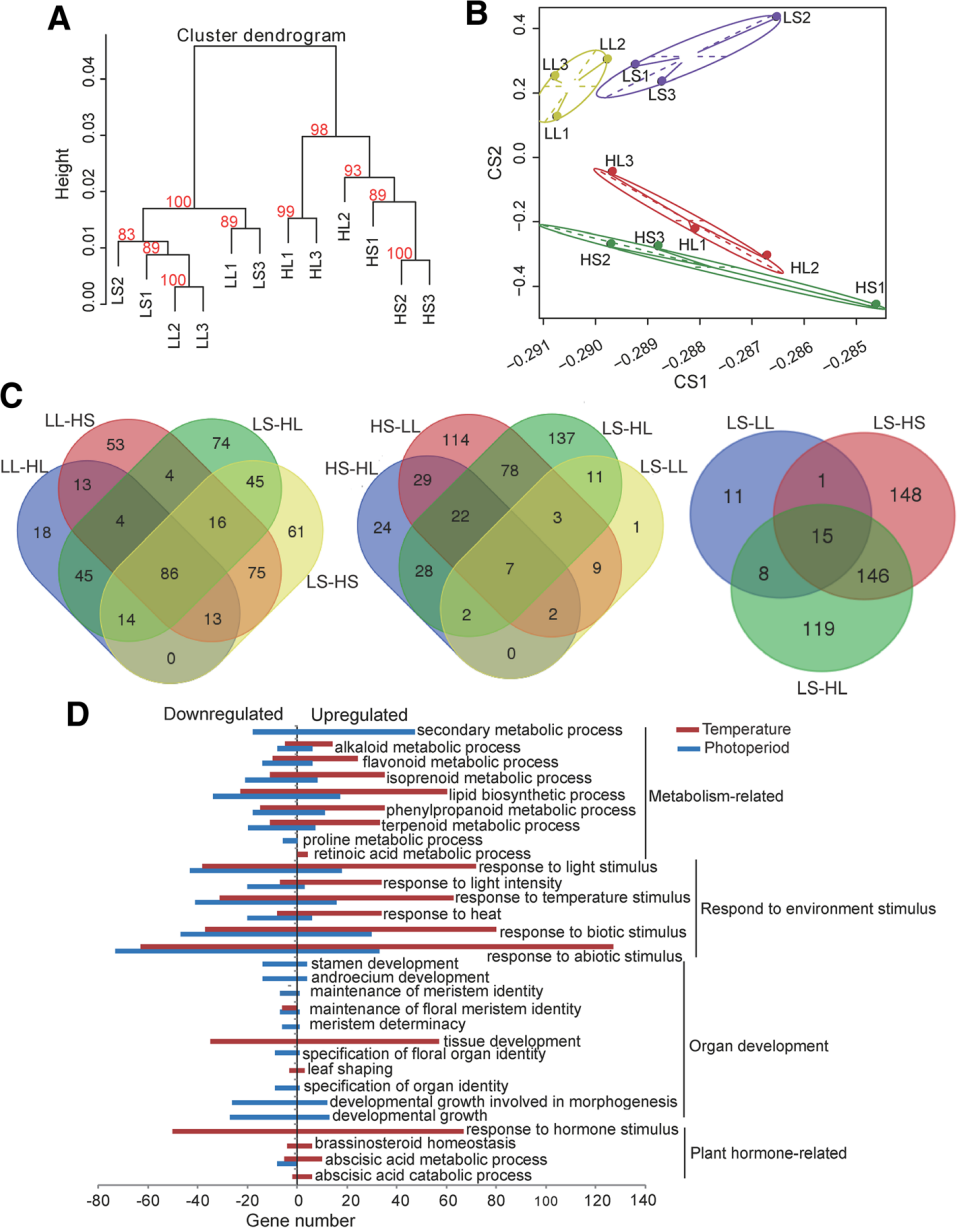
Comparisons between transcriptomes. **a** Clustering analysis of the 12 transcriptomes by a dendrogram. Approximately unbiased (AU) *P*-value is calculated by multiscale bootstrap resampling and printed in red color. **b** Two-dimensional scatter diagram of the first and second principle component (PC) scores of the transcriptomes in the principle component analysis (PCA). **c** Venn diagram of differentially expressed genes (DEGs) between temperature condition changes (left), photoperiod condition changes (middle), and environment condition changes taking LS as a control (right). LL-HL, environment condition change from LL to HL. **d** GO-process enrichment analysis of DEGs that were significantly influenced by temperature and photoperiod in ANOVA. Left and right columns indicate the number of downregulated DEGs and upregulated DEGs, respectively

We analyzed the interactions between temperature- and photoperiod-effect on the transcriptome of ‘9930’ shoot apices in the two-factor treatment. The four treatment conditions are low temperature and short day (LS), low temperature and long day (LL), high temperature and short day (HS), and high temperature and long day (HL). Clustering analyses of transcriptomes revealed that LL and LS were clustered together, and HS and HL were clustered together (Fig. 2a). In a principle component analysis (PCA), the first and the second component explained 96.9 and 1.7% of the variation, respectively. From the component plotting, a slight difference in the transcriptome clustering between long- and short-day treatment was observed while there was a large difference between the high and low temperature treatment (Fig. 2b). These results indicated that temperature is the decisive factor. Interestingly, the second principal component had a positive effect for LL and LS but a negative effect for HL and HS. Basically, there was clustering of the transcriptome triplicates, indicating good reproducibility, which is necessary for the following in silico analysis.

Many differentially expressed genes (DEGs) were shared between temperature treatments (86 DEGs), whereas only a small number were shared between the photoperiod treatments (7 DEGs) (Fig. 2c). Taking LS as a control and LL, HS, and HL as treatments, only 15 DEGs were shared among the three treatments (Fig. 2c). These results suggest distinct mechanisms for temperature- and photoperiod-related sex expression change in term of transcriptome.

A total of 537 DEGs were identified in the two-factor treatment. A two-factor ANOVA revealed that the number of genes that were significantly (*p* < 0.05) affected by temperature, photoperiod, and the interaction were 438, 244, and 192, respectively (Additional file 1: Table S3). Gene Ontology (GO) analysis of these DEGs revealed different effects on gene regulation between temperature and photoperiod (Fig. 2d). A high temperature promoted metabolism, such as lipid biosynthetic and phenylpropanoid metabolic processes, whereas long-day treatment suppressed these processes. Similarly, high temperature treatment upregulated genes that respond to environmental cues, and long-day treatment resulted in the downregulation of these genes. The greatest difference observed was for organ development and plant hormone-related processes. High temperature had profound effects on plant hormone-related processes in terms of both biosynthetic and signal transduction pathways, while long-day treatment had nearly no effect on these pathways. In contrast, long-day treatment decreased the expression of genes related to the development processes including flower organ identity and stamen development, whereas high temperature treatment had nearly no effect.

### Comparison of methylome changes in response to temperature and photoperiod treatment

**Fig. 3:**
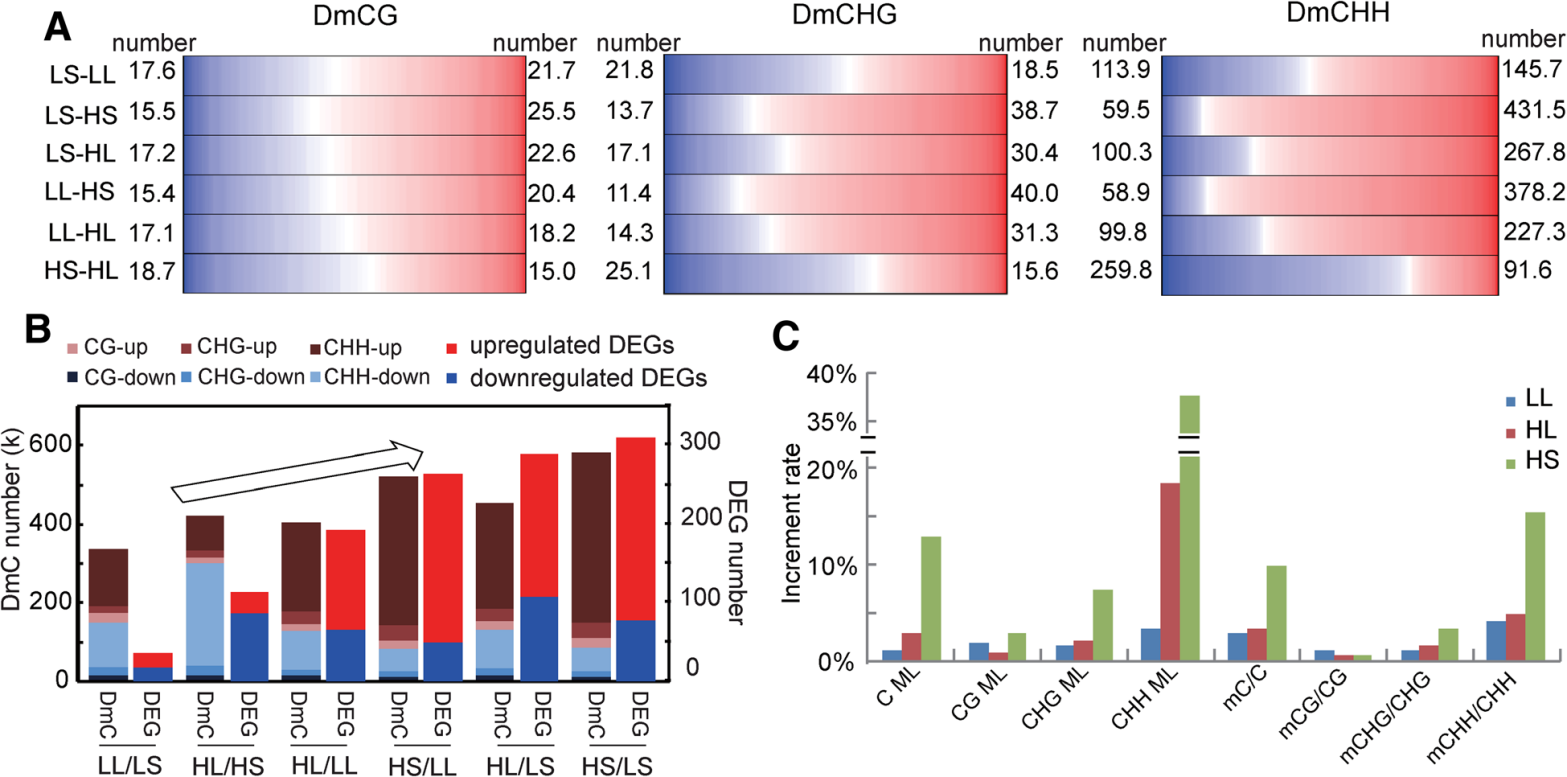
Comparisons between methylomes. **a** The number (in Million), direction and degree of methylation changes at differentially methylated cytosines (DmCs) in different condition changes. The color from red to white, the extent of the methylation change from 1 to 0; the color from blue to white, the extent of the methylation change from − 1 to 0. LS-LL, environment condition change from LS to LL. **b** The general association of differential expression and DNA methylation in the two-factor treatment. K, thousand. **c** The general change extent in methylation level (ML) and mC/C percentage resulting from the treatments of LL, HS, and HL, taking LS as a control. C/CG/CHG/CHH ML, the increment rate of methylation level of cytosine/CG cytosine/CHG cytosine/CHH cytosine

In the two-factor treatment, there is a big difference in the methylome change extent when comparing different temperature/photoperiod condition changes (Fig. 3a). The condition change of LS to HS (LS-HS) leaded to the biggest number of total differentially methylated cytosines (DmCs) (0.58 million), followed by that of LL-HS (0.52 million). While, LS-LL leaded to the least DmCs (0.34 million). For LS-HL, LL-HL, and HS-HL, the total number of DmCs ranged from 0.4 to 0.5 million. These results indicate that a temperature change seems to result in more DmCs than a photoperiod change. In plants, there are three kinds of cytosine methylation that are in contexts of CG, CHG (where H = A, T, or C), or CHH (where H = A, T, or C). Essentially, a higher temperature predominantly induced hypermethylation (LS-HS, LS-HL, LL-HS, and LL-HL); a long photoperiod predominantly induced demethylation under a higher temperature (HS-HL) whereas a slight hypermethylation under low temperature (LS-LL) (Fig. 3a). Essentially, CG showed a dynamic methylation pattern without a great preference for hypermethylation or hypomethylation compared with CHG and CHH (Fig. 3a). Notably, methylome changes were generally coordinated with transcriptome changes among the treatments (Fig. 3b). More DmCs were concomitant with more DEGs; predominant hypermethylation was concomitant with predominantly upregulated DEGs; and predominant demethylation was concomitant with predominantly downregulated DEGs.

To facilitate further study of the interaction of temperature and photoperiod, we took LS as a control and compared this condition to LL, HS, and HL in the following analysis. As a result, LL, HS, and HL all increased the extent of genomic DNA methylation in terms of both the average methylation level and methylcytosine (mC) proportion (Fig. 3c). The extent of methylation change induced by HS was greater than that induced by HL, which in turn was greater than that induced by LL. For detailed sequence types, CHH sites were common primary modification targets of all three treatments. In a detail, HS treatment resulted in an increase of CHH methylation level by 37.5% and an increase of mCHH proportion by 15.4%.

**Fig. 4:**
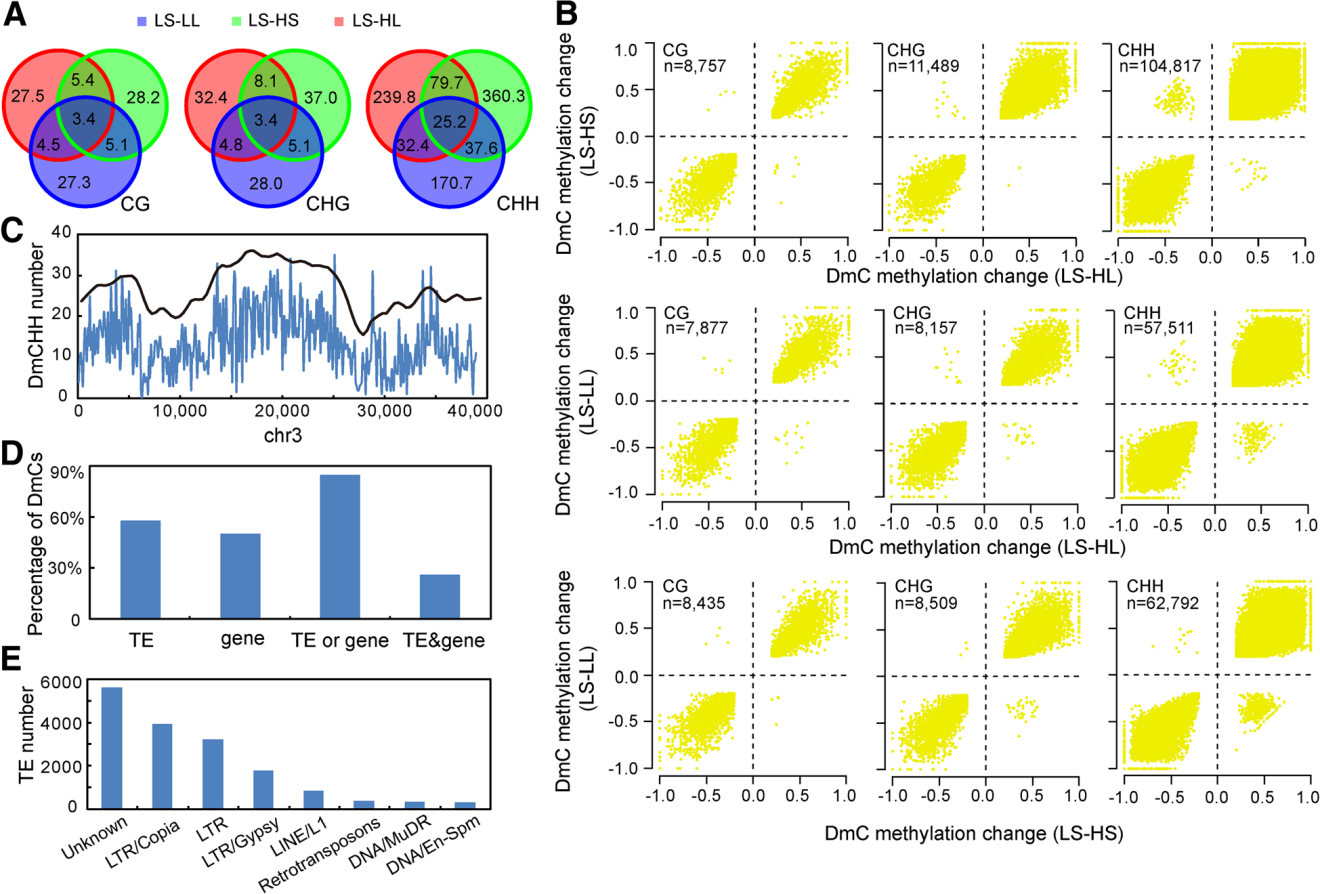
Common targeted DmCs by treatments of LL, HS, and HL, taking LS as a control. **a** Venn diagram of DmCs. The number of DmCs is indicated in thousand. **b** Correlation of the change in methylation level for common targeted DmCs. The total number of common DmCs in each two-dimensional coordinate is indicated. **c** The distribution of common targeted DmCs across chromosome 3. The black line indicates the distribution trend of mC. **d** The association of common targeted DmCs with genes and TEs. TE or gene, DmCs located in regions of genes and/or TEs; TE&gene, DmCs located in regions of genes and TEs at the same time. **e** The number of TEs for each TE type that was associated with common targeted DmCs

The proportion of DmCs shared among the three treatments (LL, HS, and HL) was small (< 4%) (Fig. 4a). Environmental treatments occasionally selected target cytosines to modify; otherwise, the proportion of common targets in different treatments should be high. However, these shared DmCs showed the same change trend (Fig. 4b). Taking together with what found in term of genomic DNA methylation extent (Fig. 3c), it is very clear that LL, HS, and HL have the same effect on cytosine methylation. Based on these results, we propose that high temperature and long day length condition both enhance cytosine methylation and temperature-effect is epistatic to photoperiod-effect. We identified 3377 CG sites, 3374 CHG sites, and 25,161 CHH sites shared among the three treatments. These cytosines showed the same distribution patterns across chromosomes as mCs (Fig. 4c) and were highly associated with transposable elements (TEs) or genes (Fig. 4d). TEs that possessed shared DmCs are likely to be the common targets in term of cytosine methylation by different environmental stimuli. Most of these TEs belonged to LTR-type followed by unknown-type and LINE-type (Fig. 4e). This constitution feature is consistent with that of the entire TEs, indicating that environmental stimuli do not have any preference about TE types when select the TE targets.

### Comparison of putative epiregulation in the temperature and photoperiod treatment

**Fig. 5:**
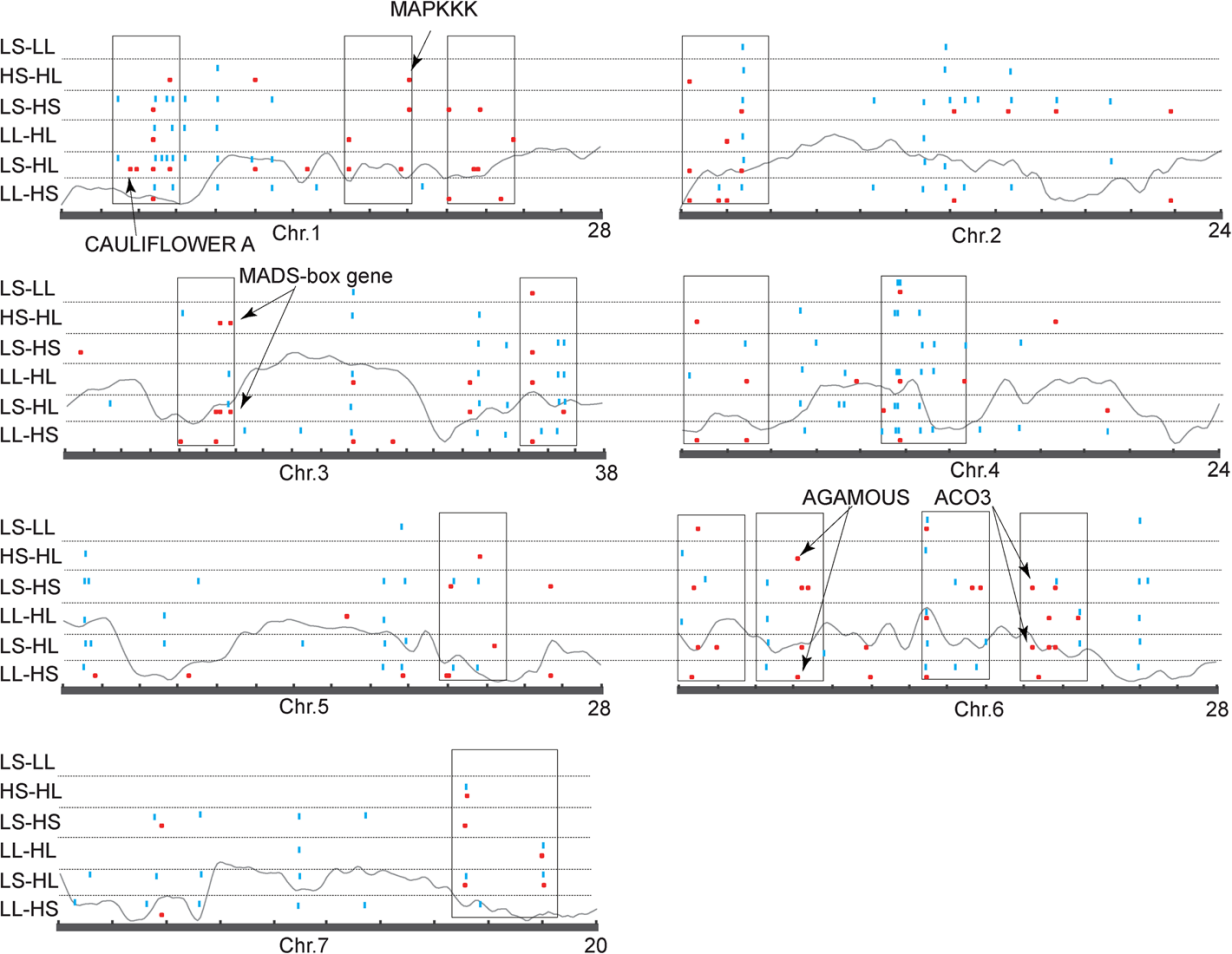
Distributions of potential epiregulation of genes (red dots) and DETs (blue dots) across chromosomes. The gray lines indicate the distribution trend of mC. *CsACO*_*3*_ gene, MADS-box genes, etc. that are proposed to be cucumber sex expression-related are shown with an arrow indicating the position. LS-LL, environment condition change from LS to LL. Boxes indicate the clustering of epiregulated regions. The nick indicates a window of 2 Mb on the chromosomes

Cytosine methylation state may affect gene activity, which is known as a kind of epigenetic regulation (epiregulation). We inferred that there was likely epiregulation determined by CG- and CHG-type differentially methylated regions (DMRs) positioned in protein-coding sequences (CDSs) and gene surrounding regions (< 2 kb from TSSs/TESs). A total of 239 association events were identified between DMRs and DEGs, among which 30.5% were positive associations. After discarding these positive associations, we proposed a total of 70 putative epiregulated DEGs. The DMRs that contributed to epigenetic control of DEGs were distributed in clusters across the genome (Fig. 5). Moreover, the occurrence of DMRs was accompanied by differentially expressed transposable elements (DETs) in most cases, although the total number of DETs was very small, suggesting a relationship between DNA methylation and TEs. Importantly, both DMRs and DETs showed high reproducibility among the different temperature/photoperiod condition changes, indicating that TE activity and DNA methylation state at some chromosome regions are highly environmentally inducible. Basically, we found a high frequency of epiregulation occurred in TE-related euchromatin regions.

Putative epiregulation sites were identified in all the temperature/photoperiod condition changes. Condition change from HS to HL (HS-HL) leads to 9 putative epiregulation sites, and LS-LL, LL-HL, LS-HS, LL-HS, and LS-HL leads to 1, 11, 20, 6, and 23 sites, respectively. Four DEGs were exclusively epicontrolled by photoperiod treatment (LS-LL and HS-HL) while the number was 11 by temperature treatment (LS-HS and LL-HL). Apparently, there were more putative epiregulation sites responding to temperature stimuli. The regulation of ethylene-related genes and MADS-box genes most likely account for cucumber sex expression change. The putative epiregulation of *CAULIFLOWER A* (Csa1M039910.1), a MADS-box transcription factor (TF) participating in flower development, was identified in LS-HL (Fig. 5). The putative epiregulation of *AGAMOUS* (Csa6M095280.1), another important MADS-box TF participating in flower development, was identified in HS-HL and LL-HS (Fig. 5). The putative epiregualtion of *CsACO*_*3*_ (Csa6M421630.1) was identified in LS-HS and LS-HL. All the above three putative epiregulations were due to CHG-type DMRs in promoters (Fig. 5).

**Fig. 6:**
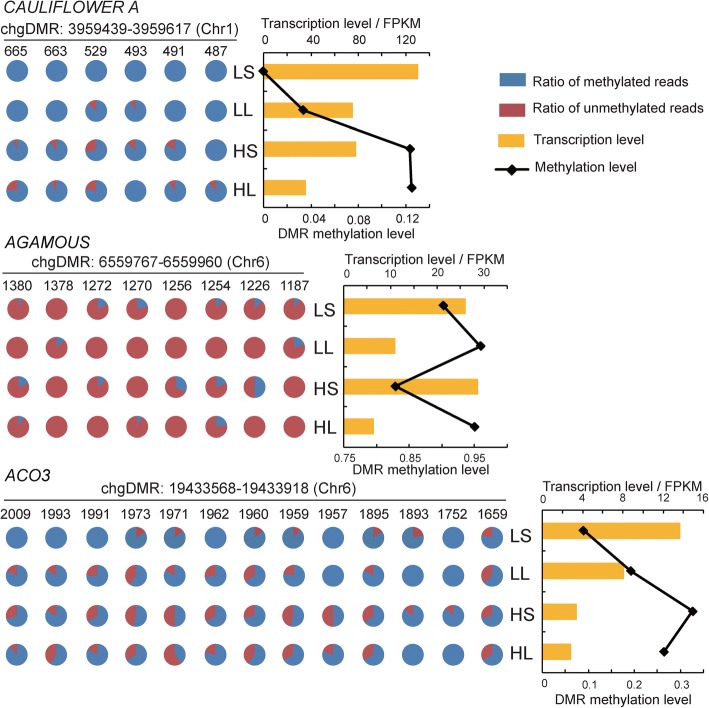
Transcription levels and promoter methylation state of MADS-box genes (*CAULIFLOWER A* and *AGAMOUS*) and *CsACO*_*3*_ gene in the treatments. Each ring represents a cytosine and the number above the rings indicates the distance to TSS site. DMR, differentially methylated regions; FPKM, fragments per kilobase of transcript per million fragments

We then thoroughly compared transcription and methylation level of MADS-box genes and *CsACO*_*3*_ gene in the temperature/photoperiod treatments. There was definitely negative association of expression level with promoter methylation level for these three genes (Fig. 6). Basically, transcription level increased along with methylation level decrease. This evidences that environment-induced DNA methylation change in promoters has an influence on gene expression. Temperature and photoperiod factors resulted in different epiregulations. *AGAMOUS* exclusively responded to photoperiod stimuli and long day treatment increased DNA methylation and decreased expression level (Fig. 6). In contrast, *CAULIFLOWER A* and *CsACO*_*3*_ were epicontrolled by both temperature and photoperiod. High temperature and long day photoperiod increased DNA methylation and decreased expression level (Fig. 6). Moreover, it is very clear that temperature-effect overwhelms photoperiod-effect, because photoperiod-effect depends on temperature condition. There results indicate temperature and photoperiod affect cucumber sex determination probably via different epicontrolled genes.

## Discussion

In this study, 359 cucumber accessions were subjected to femaleness survey in consecutive 5 years. Our studies provide consistent evidence demonstrating that season factor-dependent sex expression is a germplasm-wide phenomenon in cucumbers. There are 256 accessions, but not all of the 322 accessions showed statistical significance. This was mainly due to a technical problem in the statistical analysis. MANOVA analysis indicates a year factor that significantly affected the PNPF value and thus the year fluctuation cannot be ignored (Table 1). Moreover, many subandroecious cucumbers did not demonstrate a significant difference using the *t-test* due to a very small PNPF value and the variation source from the year factor and personal error. We give a hypothesis that all accessions except for gynoecy/subgynoecy as well as very few other accessions were sensitive to seasonal change. The multiple copy of a 30.2-kb region defines the *F* locus, which determines gynoecy expression [18], indicating a dosage effect of genetic control, which may compensate the environmental effect in gynoecious/subgynoecious lines.

Flower bud differentiation and sex expression of the first 25 nodes should have been completed within the first two or three months. The day length in March is 9.5 h and 12.67–13.87 h in April (Additional file 1: Table S2). In contrast, it is 13.70 h in mid-August, which gradually descends to 11.17 h in mid-October. Compared with spring grown cucumbers, the day length for autumn grown cucumbers is longer and the temperature is higher. The season effect must be an integration of the temperature and photoperiod effect but the change in temperature seems more drastic while the change in photoperiod is a more gradual process. Correspondingly, the normal monoecious line ‘9930’ is sensitive to both temperature and photoperiod treatments, and the temperature effect is predominant [11]. This may be a universal rule for many other monoecious cucumbers, although incubator treatment should be performed to obtain this conclusion. The change in photoperiod conditions in nature is slow and gradual and therefore, it is less of a determinant than temperature in terms of plant survival. However, the Xishuangbanna group must be an exception because these cucumbers originated in tropical area where temperature is not violently fluctuated. Our study indicates that sex expression of Xishuangbanna cucumbers is subtly regulated by photoperiod rather than temperature conditions.

Distinct mechanisms might mediate temperature and photoperiod effects on sex determination. Day length changes are sensed by circadian clocks [30]. Photoperiod conditions affect plant developmental processes, such as flowering time, hypocotyl elongation as well as female flower formation in the present study [31]. Impressively, long-day treatment significantly affected multiple development-related processes in cucumber shoot apices. A total of 18 DEGs were assigned to the GO term of “stamen development” and/or “specification of floral organ identity”, among which 15 DEGs were significantly downregulated and 8 of them were MIKC^c^ clade MADS-box genes (Additional file 1: Table S3). These MADS-box genes are probably the members of ABC model that controls floral organ identity [32], although their precise roles and functions should be clarified in the future. To date, only a very few MADS-box genes in ABC model have been experimentally identified in cucumbers. Mutation of MADS-box genes in ABC model leads to flower homeosis and thereafter sex expression in cucumbers [33]. Many flower development-related MADS-box genes were indiscriminately down-regulated in this study. Moreover, Xishuangbanna cucumber must be an extreme example showing that all flower bud differentiation is suppressed by long day condition disregarding flower sex, which implies that both female and male flowers might be affected in “9930” but unknown mechanism might relieve the effect on male flowers. Importantly, photoperiod-sensitive MADS-box genes probably also control conversion between male flowers and female flowers. CsAP3, a B-lass MADS-box transcription regulator, binds and activates the promoter of *ethylene receptor 1* (*ETR1*), a negative regulator gene of ethylene signal transduction [34]. This special association between MADS-box gene and ethylene signal transduction is only found in cucumbers, which accounts for stamen-specific downregulation of *ETR1* in female flowers [35, 36]. Temperature regulation of flower architecture involves multiple hormone signaling networks [37] and signal transduction pathways [38]. In our study, temperature stimuli got much stronger response of phytohormone-related genes than photoperiod stimuli did (Fig. 2d). Most importantly, *CsACO*_*3*_ gene was clearly downregulated by high temperature which may account for female flowers formation. In cumbers, ethylene that is the product of CsACO_3_ promotes pistil development but arrests stamen development [39].

We found that the common DmCs targeted by both high temperature and long-day treatment unexpectedly showed the same trend in changes. This result indicated that high temperature and long-day photoperiod have the same effect on methylation state at these cytosines, which is consistent with the finding that both high temperature and long-day condition suppress female flower formation. The potential epicontrol of the *CsACO*_*3*_ gene and MADS-box gene may mediate the sex expression change induced by high temperature and long-day photoperiod. Importantly, downregulation of these genes are both most likely due to CHG-type hypermethylation in the promoter regions. Since DNA methylation plays an important role in the complex process of floral transition as well as plant sex determination [40, 41], it is reasonable that environment factors affect sex expression by altering the methylation status of the regarding genes. CRISPR/Cas9-based site-specific DNA methylation modification should be a powerful tool to provide steady experimental evidences [42].

Temperature and photoperiod stimuli must share the same mechanism to reshape the DNA methylation landscape and it is highly likely that the interaction between genes and TEs is at least a part of the mechanism. Photoperiod and temperature treatments both predominantly induce CHH-type methylation changes [11]. This predominant response at CHH sequence motifs is similar to that in phosphate starvation treatment [43]. However, NaCl-saturating soil treatment [44] and bacterial infection [45] predominantly resulted in CG-type methylation changes. Environmental stimuli do not have to always have a preference in targeting CHH sites if we ignore the fact that different methods may be used in DmC and DMR identification in the above reports. CHH-type methylation is maintained and de novo is established by the RdDM pathway, indicating that the RdDM mechanism reshapes the methylome landscape in temperature and photoperiod treatments. We did not observe a clear association of 24-nt RNAs with the photoperiod treatment (data not shown), as was illustrated in the temperature treatment, likely because the photoperiod effect was much weaker than the temperature effect and therefore, was not detected. Expression-dependent RNA directed DNA methylation (RdDM), rather than the canonical RdDM mechanism, may initiate changes in DNA methylation, which can explain why environmental stimuli result primarily in the targeting of genic regions [43–45]. Expression-dependent RdDMs are triggered by Pol II mRNA transcripts rather than Pol IV transcripts and play a critical role for this type of DNA methylation [46].

## Conclusions

Seasonal changes, which are an integration of temperature and photoperiod factor, affect cucumber sex expression in a germplasm-wide manner. High temperature and long-day photoperiod, which contribute to early autumn condition, suppress female formation with different degrees of influence. A comparison among the two-factor treatment reveals that high temperature and long-day treatment have the same effect on targeted cytosines in terms of methylation state. DNA methylation-dependent epicontrol of *CsACO*_*3*_ and MADS-box genes may account for temperature- and photoperiod-induced sex expression change. Transcriptome analysis indicates that temperature and photoperiod exert an effect via phytohormone- and flower development-related genes, respectively.

## Methods

### Plant materials

In this study, a total of 359 accessions were continuously investigated over five years to ascertain the heredity of sexual type stability and the annual reproducibility. Summary information of all the cucumber materials in this study is shown in Additional file 1: Table S1. The seeds were sown on the farm at the Chinese Academy of Agricultural Sciences in Beijing in early March and in middle August every year for the spring and autumn crops, respectively. For the spring crop, cucumber seedlings were grown in a temperature- and photoperiod-controlled green house in March. Following, the seedlings were grown under natural temperature and photoperiod conditions. For the autumn crop, all materials were grown under natural temperature and photoperiod condition. Additional file 1: Table S2 shows the controlled temperature and photoperiod conditions as well as the climatological data during the survey from 2010 to 2014. The number of nodes with female flowers below the 25th node on the main stem was recorded and the intensity of femaleness is defined as proportion of nodes with pistillate flowers (PNPF). At least 15 plants were investigated for each accession each year.

A two-factor experiment was performed in incubators to study the effect of temperature and photoperiod on the femaleness of ST360 (subandroecious), ST359 (subandroecious), ST361 (gynoecious), and ‘9930’ (normal monoecious). These cucumbers are representative with different sex types. ‘BN’ originated from low-latitude southwestern China and belongs to one of the four groups known as the Xishuangbanna group (*Cucumis sativus* L. var. *xishuangbannanesis*) [28]. The four treatments were low temperature (23 °C/15 °C, day/night) and short day (8 h/16 h, day/night) (LS), low temperature and long day (16 h/8 h, day/night) (LL), high temperature (32 °C/24 °C, day/night) and short day (HS), and high temperature and long day (HL). When the fourth true leaves were unfolded, at least 30 seedlings for each accession each treatment were transplanted into a greenhouse for femaleness investigation without any temperature and photoperiod control. The femaleness survey was performed in April 2014 and the corresponding climatological data are shown in Additional file 1: Table S2.

### In silico analysis of transcriptome

The methods of sampling, RNA extraction, and RNA-seq method were reported previously [11]. Each treatment of temperature and photoperiod was conducted three times and at least 500 cucumber shoot apices were harvested each time under a microscope for following RNA extraction and RNA-seq. Clean reads were mapped to the Chinese Long *Cucumis sativus* genome version 2.0 (version 2.0; http://cmb.bnu.edu.cn/Cucumis_sativus_v20/) using BWA [47]. The transcripts were annotated after assembling by referring to the annotation file “Cucumber_v2.gff3” (version 2.0; http://cmb.bnu.edu.cn/Cucumis_sativus_v20/). The transcript levels were calculated as fragments per kilobase of transcript per million fragments (FPKM) using the Cufflinks software package [48]. Differentially expressed genes (DEGs) were identified using the NOIseq package, and the criteria were a divergence probability≥0.8 and a fold-change≥2 [49, 50]. We normalized TE transcripts to the total number of reads aligned for each TE and this value was expressed as FPKM [51]. Adjusted *p*-values (< 0.05) were used to determine the statistical significance of differentially expressed TEs (DETs). Transposons that overlapped with protein-coding genes were discarded.

### In silico analysis of methylome

The methods of sampling, RNA extraction, and RNA-seq method were reported previously [11]. Each treatment of temperature and photoperiod was conducted three times and at least 500 shoot apices were harvested under a microscope each time. All the shoot apices from each treatment were then pooled for the following DNA extraction and whole-genome bisulfite sequencing (WGBS). Methylcytosines (mCs) were identified as previously described [52]. The number of methylation-supporting reads of an mC was required to be at least the anticipated number in a binomial test adjusted by the BS conversion rate. Identification of differentially methylated cytosine (DmCs) between two treatments was performed using *Fisher’s exact test*. Cytosine sites with a *p-*value< 0.05 and changes in methylation levels of at least 20% were identified as DmCs. Differentially methylated region (DMRs) were identified as previously described [11]. Briefly, five adjacent CG/CHG/CHH motifs containing at least four CG/CHG/CHH sequences with the same response pattern and Wilcoxon rank-sum test *p*-values< 0.05 were considered candidate DMRs. Next, 3′ downstream adjacent CG/CHG/CHH sequences with the same response patterns were incorporated with the candidate DMR until the differential significance disappeared. DMRs that were smaller than 50 bp in length and for which the methylation level differences were smaller than 0.1 were discarded.

### Association between genes, TEs, and DMRs

The position of TEs and gene structures was determined by referring to the cucumber genome annotation (version 2.0; http://cmb.bnu.edu.cn/Cucumis_sativus_v20/). TSS and TES are simply determined as the boundary of an mRNA in the annotation. A genic region includes the gene body and the surrounding 2-kb regions (Fig. 4d). A TE includes only the body. A C/mC/DmC is allowed to be collated in more than one genomic feature, e.g.*,* TEs, genic regions. The position of a DMR relative to genic regions determined relative to the midpoint of each DMR; the association of a DMR with a TE was confirmed if the regions overlapped; the association of TE and genic regions was confirmed if they overlapped. Each genic DMR was only assigned to the nearest genes. The position of DMR-DEG associations and DETs was determined relative to the midpoint of each DMR and DET (Fig. 5).

### Statistical analysis

The biological variables of cucumber PNPF values are not normally distributed (Additional file 2: Figure S1). To meet the assumptions of the parametric statistical test, all of the PNPF values were normalized by arc sine transformation prior to Multivariate Analysis of Variance (MANOVA). The normalized value = asin(sqrt(PNPF)). MANOVA of the femaleness survey in cucumber germplasm was performed using SAS/STAT® software (SAS 9.2, SAS Institute Inc., Cary, NC, USA).

Analysis of Variance (ANOVA) analyses were performed to study the effect of temperature and photoperiod on femaleness as well as gene expression. All ANOVA analyses were performed using the SPSS statistic software (SPSS 20.0, SPSS Inc., Chicago, IL, USA).

### Data access

Whole-Genome Bisulfite Sequencing (WGBS) data were retrieved from NCBI SRA with the accession ID to be SRR5430777 (HS), SRR5430103 (LS), SRR5430207 (LL), and SRR5431155 (HL). Transcriptome sequencing data were retrieved from NCBI SRA database. The accession ID of three HS replicates is SRR5462513, SRR5462516, and SRR5462554; that of three LS replicates is SRR5460753, SRR5461296, and SRR5461309; that of three LL replicates is SRR6837824, SRR6837841, and SRR6837842; that of three HL replicates is SRR6837906, SRR6837907, and SRR6837908.

## Additional files

Additional file 1: **Table S1.** Summary of the cucumber materials. **Table S2.** The temperature and photoperiod condition in the investigation of seasonal change of cucumber PNPF value. **Table S3.** Double factor variance analysis. (XLSX 105 kb)

Additional file 2: **Figure S1.** Histograms of number of NRPF at different levels. (A) Untransformed data. (B) arc sine-transformed data. (TIF 894 kb)

## Abbreviations

CDS: Protein-coding sequence
DEG: Differentially expressed gene
DET: Differentially expressed transposable element (TE)
DmC: Differentially methylated cytosine
DMR: Differentially methylated region
HL: High temperature and long day
HS: High temperature and short day
LL: Low temperature and long day
LS: Low temperature and short day
mC: Methylcytosine
PNPF: Proportion of nodes with pistillate flowers
RdDM: Small RNA-directed DNA methylation
TES: Transcription end site
TSS: Transcription start site
WGBS: Whole-genome bisulfite sequencing
