# Supplementary figures and images for: Temperature and photoperiod changes affect cucumber sex expression by different epigenetic regulations
Source: BMC Plant Biol. 2018 Nov 6;18:268. doi: 10.1186/s12870-018-1490-3 (PMC6220452; doi:10.1186/s12870-018-1490-3)

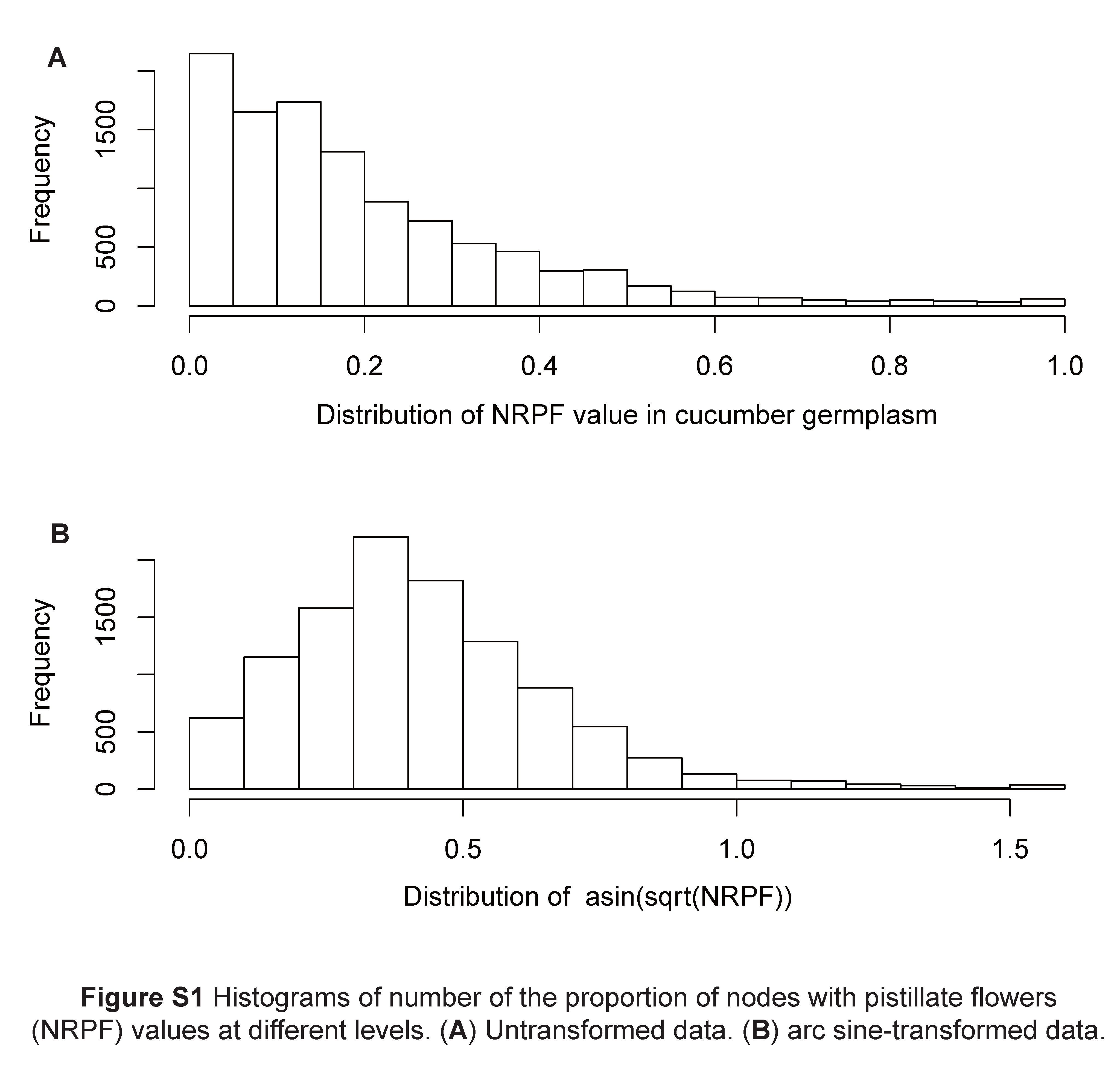

Supplement: Supplementary file 2 — Figure S1. Histograms of number of NRPF at different levels. (A) Untransformed data. (B) arc sine-transformed data. (TIF 894 kb) [file 12870_2018_1490_MOESM2_ESM.tif]
